# Mathematical modeling of water sorption isotherms in specialty coffee beans processed by wet and semidry postharvest methods

**DOI:** 10.1038/s41598-024-83702-y

**Published:** 2025-01-31

**Authors:** Gentil A. Collazos-Escobar, Valeria Hurtado-Cortés, Andrés Felipe Bahamón-Monje, Nelson Gutiérrez-Guzmán

**Affiliations:** 1 Centro Surcolombiano de Investigación en Café (CESURCAFÉ), Departamento de Ingeniería Agrícola, Universidad Surcolombiana, 410001 Neiva-Huila, Colombia; 2 Grupo de Análisis y Simulación de Procesos Agroalimentarios (ASPA), Instituto Universitario de Ingeniería de Alimentos–FoodUPV, Universitat Politècnica de València, Camí de Vera S/N, Edificio 3F, 46022 Valencia, Spain

**Keywords:** Hygroscopicity, Machine learning, Optimization, Storage management, Shelf life, Moisture stability, Chemistry, Engineering

## Abstract

This study investigates the experimental assessment and mathematical modeling of the water sorption isotherms in dried specialty coffee beans processed by wet and semidry postharvest methods. The wet and semidry sorption isotherms were experimentally obtained over a range of water activities between 0.1 and 0.85 at temperatures of 25, 35, and 45 °C using the dynamic dew point method (DDI). Mathematical modeling was conducted to describe the influence of water activity, temperature, and postharvest method on the equilibrium moisture content. Twelve conventional sorption equations and four machine learning techniques were employed for modeling, using 75% of the experimental data for training and 25% for validation. The selection of the best model was carried out via multifactor Analysis of Variance (ANOVA). Experimental results showed that wet and semidry coffee beans exhibited a type II S-shaped isotherm (Brunauer–Emmett–Teller classification) and a significant (*p* < 0.05) influence of temperature on sorption curves. Additionally, the mucilaginous coating found in semidry coffee beans provided a protective role against water sorption. The Support Vector Machine (SVM) model provided the best fit for describing the sorption isotherms (mean relative error, MRE < 1% and adjusted coefficient of determination, *R*^2^_adj_ > 99%), demonstrating its robustness in predicting the equilibrium moisture content as a function of water activity, temperature, and postharvest processing method. This mathematical model could serve as a virtual representation of the storage process, facilitating real-time decision-making to enhance coffee quality management during storage.

## Introduction

Coffee is one of the most important agricultural commodities for the economies of producing countries^1^. Coffee-based products are recognized globally for their unique sensory profile and stimulating properties^2^. In recent years, the coffee industry has undergone significant changes with the emergence of specialty coffee^3^ and growing consumer concern for high-quality coffee beans produced in environmentally sustainable ways. This trend has influenced both production practices and market preferences, placing increased emphasis on quality and sustainability^4^.

Currently, a substantial portion of the world’s coffee supply is processed using the wet postharvest method, which remains the dominant approach due to its ability to enhance the desired characteristics of coffee beans. In fact, during the 2019/2020 season, global coffee production reached approximately 846,000 tons (ICO, 2023), most of which relied on this postharvest method. The massive volume of coffee produced and its increasing demand also rise its environmental impact, creating severe problems in the producer countries, including higher water consumption and pollution of both water resources and soil^5^. Therefore, exploring new, more sustainable, and environmentally friendly coffee processing strategies is crucial. Alternative semidry postharvest methods have been proposed to address these challenges in producing specialty coffee. The coffee beans processed by this method reduce their impact on water usage while still producing high-quality coffee with a distinctive and differentiated flavor and aroma^6^.

As coffee producers become increasingly aware of the environmental damage caused by the traditional wet processing method and the challenges faced in achieving sustainability within the coffee industry, many are gradually adopting the semidry method as an alternative processing technique to obtain specialty coffees. However, there is limited information on correctly processing and managing the coffee obtained by this method, particularly regarding storage. Furthermore, there is a significant gap in the literature concerning the adequate storage of semidry coffee to guarantee its shelf life and quality. Even though coffee processed by the wet method has been extensively studied^7–9^. In this sense, the study of interactions between both temperature and relative humidity (%ERH) and coffee beans during storage is indispensable to achieve this goal^10^.

The water sorption isotherms describe the relationship between the equilibrium moisture content (X_e_) and water activity (a_w_, %ERH/100) of products and their interaction with the surrounding conditions of temperature and ERH during storage^11,12^_._ Therefore, water sorption isotherms could be considered as a valuable tool for selecting appropriate packaging material and in determining foodstuff storage conditions to minimize spoilage reactions affecting the product’s texture, aroma, flavor, or color^13,14^. The water sorption isotherms for dried specialty coffee processed using the semidry method have not yet been determined. Moreover, no comparison has been made between the wet (traditional) and semidry (alternative method for reducing water usage) processing methods. Understanding these isotherms provides reliable information for the coffee industry, enabling the optimization of storage conditions to minimize quality degradation.

Traditionally, the water sorption isotherms are gravimetrically determined using the saturated salt slurries method. Several issues in this method include the limited data points obtained in every test, the slow speed analysis, and mold growth risks in the food samples at higher a_w_ levels (a_w_ > 0.7)^15^. These issues complicate the rapid analysis of water sorption properties of food products and constrained its further mathematical modeling for making predictions of moisture content as a function of a_w_ and temperature. In this way, developing dynamic dew point (DDI) instruments overcomes these challenges. The DDI instrument operates by directly measuring the a_w_ of samples using cooled mirror technology, allowing for precise a_w_ values without the need for prior equilibration at a specific ERH. As weight changes are monitored gravimetrically, the DDI facilitates the generation of water sorption isotherms by quantifying vapor adsorption and desorption in real-time. This approach enables the collection of extensive data points across a wide range of conditions in a significantly shorter timeframe^16^. Several works have reported the feasibility of the DDI method for determining accurate and precise water sorption isotherms of coffee beans and food products such as cherry coffee beans^17^, green and roasted coffee beans^7^, roasted/ground specialty coffee, Achira biscuits^18^, maize byproducts^19^ and dry-crystallized *Palada payasam*^20,21^.

Mathematical modeling of water sorption isotherms constitute a powerful tool for a better understanding the food-surrounding interaction during storage. The calibrated models could be used for predicting and simulating changes in food moisture content at different temperatures and a_w_ conditions, providing the basis for supporting real-time decision-making in the coffee industry^22^. Conventional models used for describing the water sorption isotherms include from theoretical (Guggenheim-Anderson-de Boer; GAB and Brunauer–Emmett–Teller; BET) to empirical (Oswin, Smith, Henderson, Halsey, Kuhn, among others)^23^. However, given the complexity of food products and the multivariate nature of the actual food processing, it is essential to address new computer-aided data-driven models that could be able to describe not only the influence of temperature and a_w_ over moisture content but also the influence of variables related to the food manufacturing processes (in the case of coffee: different coffee varieties, postharvest processing activities such as fermentation, drying, storage, roasting, among others). Thus, machine learning (ML) techniques, including Regression Trees (RT), Random Forest (RF), k-Nearest Neighbors (kNN), and Support Vector Machines (SVM) have demonstrated their potential in solving multidimensional modeling problems in the coffee industry^24–27^.

To our knowledge, a robust computer model that addresses conventional sorption models and machine learning techniques has not been extensively explored for water sorption isotherms in specialty coffee beans processed using different postharvest methods. The coffee industry could significantly benefit from developing these tools, which would support decision-making and facilitate process improvements in the storage and management of specialty coffee. Considering the aspects mentioned above, this work aims to experimentally determine the water sorption isotherms of dried specialty coffee beans obtained by wet and semidry postharvest methods at 25, 35, and 45 °C using the DDI method, and to assess computer-aided mathematical modeling of water sorption isotherms using both conventional GAB and empirical sorption models, and a novel approach based on machine learning techniques to describe the influence of the a_w_, temperature and postharvest method on the X_e_.

## Materials and methods

### Samples

**Fig. 1:**
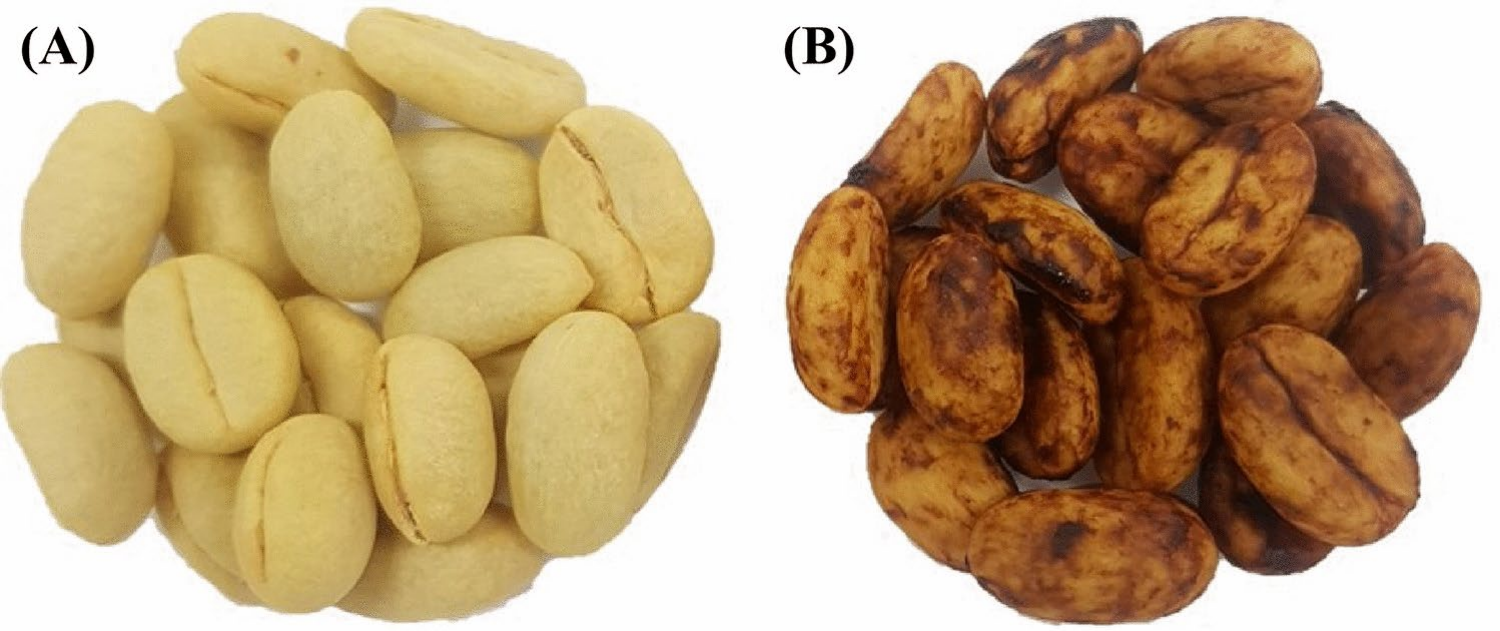
Parchment specialty coffee beans obtained by wet (**A**) and semidry (**B**) methods.

Eighteen coffee samples (20 kg each) of the Bourbon Rosado variety (*Coffea arabica* L.) were kindly provided by various farmers from San Adolfo-Huila region of Colombia (coordinates: 1° 42′ 4′′ N, 76° 01′ 32′′ W, at an altitude of 1664 m above sea level)., who granted permission for the use of these private cultivars. The samples were transported in refrigerated coolers at 4 °C to the Centro Surcolombiano de Investigación en Café (CESURCAFÉ, Neiva-Huila, Colombia) for processing using wet and semi-dry methods. For the wet method (Fig. 1A), the coffee samples were pulped (Gaviota 300, Ingesec, Colombia), fermented in plastic containers for 18 h, and washed to eliminate excess mucilage^3^. The coffee beans were pulped and dried immediately in the semidry method (Fig. 1B). Coffee samples from both postharvest methods were sun-dried^28^ daily between 10 a.m. and 5 p.m. at temperatures of 35 ± 3 ºC and 15–45%ERH. The moisture content of coffee samples was monitored using a handheld portable grain moisture tester (Kett PM − 450, Science of Sensing, Japan) every 1 h during the sun-dry process. This step was carried out until the beans reached a moisture content of 9–11% (wet basis, %w.b.). Afterward, the dried coffee samples were sensory analyzed using the Specialty Coffee Association methodology^29^ at the CESURCAFÉ. The cup scores were between 80.25 and 86, so the samples were considered high-quality specialty coffee.

### Initial moisture content and water activity

The initial moisture content of the dried coffee beans was determined by drying 10 g of sample in an oven (UF55, Memmert GmbH + Co. KG, Schwabach, Germany) at 105 °C for approximately 24 h until the weight of the coffee beans was constant. The a_w_ was determined using a vapor sorption analyzer (VSA Aqualab Decagon Devices, Inc. Pullman, WA). In each trial, 5.0 g of sample was used and the analysis was conducted in triplicate for both moisture content and a_w_.

### Experimental determination of water sorption isotherms

The experimental water sorption isotherms were obtained using the dynamic dewpoint isotherm (DDI) method with a VSA instrument (Aqualab, Decagon Devices, Inc., Pullman, WA). In each test, 4 to 5 g samples of dried specialty coffee beans obtained by wet and semidry methods were placed inside the VSA instrument. Since the samples had an intermediate value of a_w_ (approximately 0.6 a_w_^30^), they were subjected independently to a desorption test within the range of 0.6 to 0.1 a_w_ and adsorption tests from 0.6 to 0.85 a_w_. These measurements were conducted in triplicate at temperatures of 25, 35, and 45 °C, with an interval of 0.01 a_w_ for each measurement and a water vapor airflow rate of 100 mL min^–1^. The desorption and adsorption isotherms from each trial were combined to obtain the water sorption isotherm for each sample.

### Mathematical modeling

#### GAB and empirical models

The water sorption isotherms were mathematically represented using twelve equations (Table 1, Eqs. 1 to 14) commonly cited in the literature for describing the influence of a_w_ and temperature on the X_e_^12,31–35^. Since the water sorption isotherms of specialty coffee beans processed by semidry postharvest methods have not been previously reported in the literature, and no single model has been established to describe the sorption behavior of all agricultural products, it is essential to evaluate the performance of various models in accurately describing sorption isotherms, especially when conducting new analyses of specific food commodities^1^. Thus, the mathematical models considered for fitting water sorption isotherms included the theoretical GAB model (Eqs. 1 to 3), as well as the empirical models by Peleg (Eq. 4), Smith (Eq. 5), Kuhn (Eq. 6), Double Logarithm Polynomial (DLP, Eq. 7), Chung-Pfost (Eq. 8), Caurie (Eq. 9), Iglesias & Chirife (Eq. 10), White & Eiring (Eq. 11), Polynomial (Eq. 12), Oswin (Eq. 13), and Yanniotis & Blahovec (Eq. 14). A nonlinear ordinary least squares (OLS) regression approach was employed to fit the models by minimizing the residual mean square (RMS, Eq. 15). The statistical estimation of the model parameters was performed by using MATLAB® R2023a (The MathWorks, Inc., Natick, MA, USA).

15 $$\text{RMS}=\frac{\sum_{\text{i}=1}^{\text{N}}{\left({\text{Y}}_{\text{exp}}- {\text{Y}}_{\text{cal}}\right)}^{2}}{\text{N}}$$

where Y_exp_ is the experimental response variable, Y_cal_ is the predicted response, and N is the number of experimental data points. The initial parameters (X_m_, C, and K, Table 1) of the GAB model were determined by rearranging Eq. 1 into a second-order polynomial expression (Eq. 16). The calculation process is outlined below.

**Table 1:** Mathematical models used to describe the water sorption isotherms of specialty coffee beans.

Model	Mathematical expression	References	Eq
GAB	${\text{X}}_{\text{e}}=\frac{{\text{X}}_{\text{m}}\text{ CK}{\text{a}}_{\text{w}} }{\left(1-{\text{Ka}}_{\text{w}}\right)(1+(\text{C}-1){\text{Ka}}_{\text{w}})}$	1	1
	$\text{C}={\text{C}}_{0}\text{ exp}(\frac{{\text{H}}_{\text{m}}-{\text{H}}_{\text{n}}}{\text{RT}})$		2
	$\text{K}={\text{K}}_{0}\text{ exp}(\frac{\uplambda -{\text{H}}_{\text{n}}}{\text{RT}})$		3
Peleg	${\text{X}}_{\text{e}}={\text{a}}_{0}{{\text{a}}_{\text{w}}}^{{\text{a}}_{1}}+{\text{a}}_{2}{{\text{a}}_{\text{w}}}^{{\text{a}}_{3}}$	12	4
Smith	${\text{X}}_{\text{e}}={\text{a}}_{1}-{\text{a}}_{2}\text{ln}(1-{\text{a}}_{\text{w}})$	12	5
Kuhn	${\text{X}}_{\text{e}}=\left(\frac{{\text{a}}_{1}}{{\text{lna}}_{\text{w}}}+{\text{a}}_{2}\right)$	31	6
DLP	${\text{X}}_{\text{e}}={\text{a}}_{0}+{\text{a}}_{1}\text{x}+{{\text{a}}_{2}\text{x}}^{2}+{{\text{a}}_{3}\text{x}}^{3}$ $\text{x}=\text{ln}(-{\text{lna}}_{\text{w}})$	40	7
Chung-Pfost	${\text{X}}_{\text{e}}={\text{a}}_{1}-{\text{a}}_{2}\text{ln}(-{\text{lna}}_{\text{w}})$	34	8
Caurie	${\text{X}}_{\text{e}}=\text{exp}({\text{a}}_{1}+{\text{a}}_{2}{\text{a}}_{\text{w}})$	30	9
Iglesias & Chirife	${\text{X}}_{\text{e}}={\text{a}}_{1}+{\text{a}}_{2}(\frac{{\text{a}}_{\text{w}}}{1-{\text{a}}_{\text{w}}})$	30	10
White & Eiring	${\text{X}}_{\text{e}}=\frac{1}{{\text{a}}_{1}+{\text{a}}_{2}{\text{a}}_{\text{w}}}$	30	11
Polynomial	${\text{X}}_{\text{e}}={\text{a}}_{0}+{\text{a}}_{1}{\text{a}}_{\text{w}}+{{\text{a}}_{2}{\text{a}}_{\text{w}}}^{2}+{{\text{a}}_{3}{\text{a}}_{\text{w}}}^{3}$	40	12
Oswin	${\text{X}}_{\text{e}}={\text{a}}_{1}{\left(\frac{{\text{a}}_{\text{w}}}{1-{\text{a}}_{\text{w}}}\right)}^{{\text{a}}_{2}}$	31	13
Yanniotis & Blahovec	${\text{X}}_{\text{e}}=\left(\frac{{\text{a}}_{\text{w}}}{{\text{a}}_{0}+{\text{a}}_{1}{\text{a}}_{\text{w}}}\right)+(\frac{{\text{a}}_{\text{w}}}{{\text{a}}_{2}+{\text{a}}_{3}{\text{a}}_{\text{w}}})$	21	14

16 $$\frac{{\text{a}}_{\text{w}}}{{\text{X}}_{\text{e}}}=\frac{1}{{\text{X}}_{\text{m}}\text{C K}}+\frac{\text{C}-2}{{\text{X}}_{\text{m}}\text{C}}{\text{a}}_{\text{w}}+\frac{\text{K}(1-\text{C})}{{\text{X}}_{\text{m}}\text{C}}{\text{a}}_{\text{w}}^{ 2}$$

The empirical (a_i_) model parameters (Table 1) were initialized using a genetic algorithm (GA), which was computed using the “ga” MATLAB function^18^. Subsequently, both the GAB and a_i_ terms were optimized for each experimental temperature using the “nlinfit” MATLAB function, and the confidence intervals (95%) of these parameters were estimated with the “nlparci” MATLAB function.

Afterward, both Arrhenius expressions (Eqs. 2 and 3) were linearized to link the influence of temperature on the C and K parameters. Thus, the remaining GAB parameters were estimated, such as C_0_, K_0_, H_m_ (kJ kg^−1^), and H_n_ (kJ kg^−1^). Furthermore, the latent heat of water vaporization (λ, kJ kg^−1^) was also considered for this calculation.

To determine the effect of temperature on the a_i_ parameters, a linear dependence was considered^36^. Furthermore, to obtain a robust model to describe both the water sorption isotherms considering the temperature effect, the differences between the desorption and adsorption isotherms (as already explained in the experimental determination of water sorption isotherms section) and the influence of postharvest methods, two dummy variables (D_i_) were used for this purpose. Each dummy variable was associated with the GAB model and empirical expressions using a regression coefficient (b_i_), as explained below by an example using the GAB model (Eq. 17).

17 $${\text{X}}_{\text{e}}=\frac{{\text{X}}_{\text{m}}\text{ CK}{\text{a}}_{\text{w}} }{\left(1-{\text{Ka}}_{\text{w}}\right)(1+(\text{C}-1){\text{Ka}}_{\text{w}})}+{\text{b}}_{1}{\text{D}}_{1}+{\text{b}}_{2}{\text{D}}_{2}$$

where D_1_ is a dummy variable whose 0 value indicates the reference wet method, 1 indicates the semidry process, D_2_ is a dummy variable whose 0 value indicates the reference desorption isotherm and 1 indicates the adsorption isotherm. These regression coefficients (b_1_ and b_2_) quantify the changes in X_e_ (% d.b.) between reference situations and positive situations, and they were found by minimizing the RMS procedure, as previously mentioned.

### Supervised machine learning techniques

Four machine learning (ML) techniques, namely, Regression Trees (RT), Random Forest (RF), k-Nearest Neighbors (kNN), and Support Vector Machines (SVM), were considered for the mathematical description of water sorption isotherms. In this case, the ML techniques used regressor variables such as a_w_, temperature, the type of isotherm (desorption/adsorption), and the postharvest method to describe their influence on the X_e_. The computer-aided procedure involved nonlinear OLS regression through minimization of the RMS (Eq. 15).

The ML algorithms were computed using the R Core Team’s statistical software (2023), as explained below. The assessment of RT was performed using a post-pruning standard error of 0.1, and the computational procedure was conducted using the *rpartXse* function of the *DMwR2* R package^37^. The RF calculations using 100 trees were carried out via the *randomForest* function via the *randomForest* R package^38^. The Fast Nearest Neighbor, considering four nearest neighbors was achieved by employing the *knn* function of the *FNN* R package^39^. Moreover, the SVM was computed considering a *Laplacedot* kernel function, *nu-SVR* type, and C parameter equal to 500.5. Vector machines were appraised using the *ksvm* function of the *kernlab* R package^40^. These R functions and packages were used due to their open-source accessibility and reproducibility for fitting advanced machine learning models such as RT, RF, kNN, and SVM.

### Model training and statistical validation

The experimental water sorption isotherms were randomly partitioned 100 times into two datasets: one segment for model training (75%) and the remaining (25%) for the statistical validation of the trained model^41^. To calculate the predictive ability of the trained and validated models, different goodness of fit metrics, such as the mean relative error (MRE) (Eq. 18) and the adjusted coefficient of determination (R^2^_adj_) (Eq. 20), were considered.

18 $$\text{MRE }\left(\text{\%}\right)=\frac{100}{\text{N}}\sum_{\text{i}=1}^{\text{N}}\frac{\left|{\text{Y}}_{\text{exp}}- {\text{Y}}_{\text{cal}}\right|}{{\text{Y}}_{\text{exp}}}$$

19 $${\text{R}}^{2}(\text{\%})=100-\frac{\sum_{\text{i}=1}^{\text{N}}{\left({\text{Y}}_{\text{exp}}- {\text{Y}}_{\text{pred}}\right)}^{2}}{\sum_{\text{i}=1}^{\text{N}}{\left(\overline{{\text{Y} }_{\text{exp}}}- {\text{Y}}_{\text{pred}}\right)}^{2}}$$

20 $${\text{R}}_{\text{adj}}^{2}(\text{\%})=100-\left(\frac{\text{N}-1}{\text{N}-\text{M}}\right)\left(100-{\text{R}}^{2}\right)$$

where M is the number of parameters in the models and R^2^ is the coefficient of determination between the experimental data and their values predicted by the mathematical models.

### Statistical analysis

To validate the trained models, their residuals were summed for nonrandom and heteroscedastic tests^19^. Hence, Ljung-Box and Levene’s tests were performed to test these hypotheses (*p* < 0.05). Additionally, to select the best-trained model for representing the experimental validation dataset (25%), a multifactor analysis of variance (ANOVA) was performed considering the mathematical models and iterations (100 times) as factors and both MRE(%) and R^2^_adj_(%) as response variables. The comparative means pairwise coupled with Fisher’s least significant difference (LSD) intervals (*p* < 0.05) were used to determine whether the factors above significantly influenced the mean values of MRE and R^2^_adj_. Residual validation of the multifactor ANOVA model was performed by verifying the residuals’ normality, independence, and homoscedasticity. The Shapiro–Wilk test and q-q plot were used to evaluate residual normality, while the Ljung–Box test was employed to check for residual independence. Multiple linear regression (MLR) was applied to the squared residuals to examine homoscedasticity to detect any significant patterns or deviations. All hypothesis tests and statistical assumptions were evaluated at a 95% confidence. The statistical analysis was conducted using STATGRAPHICS Centurion XVIII (Manugistics, Inc., Rockville, MD, USA).

## Results

### Experimental water sorption isotherms

**Fig. 2:**
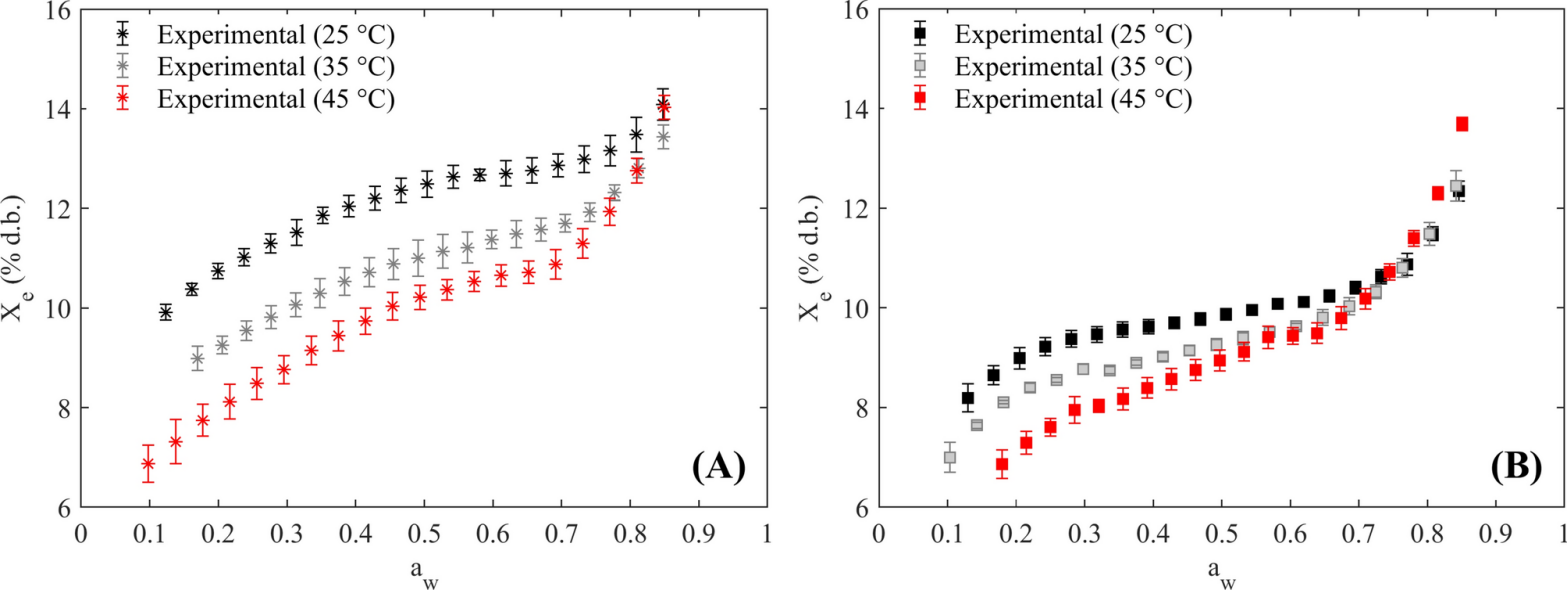
Experimental water sorption isotherms of coffee processed via the wet method (**A**) and the semidry method (**B**) at temperatures of 25, 35, and 45 °C.

The water sorption isotherms as a function of the experimental temperature for specialty coffee beans obtained via wet and semidry postharvest methods are illustrated in Fig. 2.

The water sorption isotherms for both types of coffee beans displayed a sigmoid S-shape, corresponding to Type II in the BET classification, which is typical of macroporous food products^42^. The X_e_ increased with rising a_w_ was found_,_ while the higher experimental temperatures led to decrease in X_e_^34^. This trend was observed within the a_w_ range of 0.1 and 0.8 for water sorption isotherms obtained by the wet method and 0.1 to 0.7 a_w_ for the semidry. Additionally, the temperature influence was not statistically significant (*p* > 0.05) at a_w_ levels higher than 0.8 in the wet method. Conversely, in the semidry processing, the increase in temperature promoted an increase in the X_e_ (crossing phenomenon)^31,32,43^ in a_w_ values above 0.7. Differences between the S-shaped type II isotherms were evident for wet and semidry methods and the effect of temperature on the water sorption isotherms was more pronounced in the coffee samples processed by the wet method.

### Mathematical modeling of water sorption isotherms

The statistical results of the mathematical modeling of water sorption isotherms, as a function of the postharvest method, obtained through the computer-aided procedure using the GAB model, empirical models, and machine learning techniques, are presented separately for the training (75%) and validation (25%) data sets in Tables 2 and 3.

**Table 2:** Estimated model parameters of the trained models (75%) and their statistical results using the validation dataset (25%).

Models	Parameters	Confidence intervals (95%)	MRE (%)	R^2^_adj_ (%)
Iglesias & Chirife	a_1.1_ = –1.10 × 10^–3^ K^–1^ a_1.2_ = 0.44 a_2.1_ = 2.5 × 10^–4^ K^–1^ a_2.2_ = –0.07 b_1_ = –0.02% d.b b_2_ = –2.6 × 10^–4%^ d.b	[–1.18 × 10^–3^, –1.06 × 10^–3^] [0.43, 0.46] [2.3 × 10^–4^, 2.9 × 10^–4^] [–0.08, –0.06] [–0.02, –0.02] [–1.4 × 10^–3^, 9.4 × 10^–4^]	Training 4.5 ± 0.1 Validation 4.5 ± 0.2^aA^	Training 93.7 ± 0.1 Validation 93.6 ± 0.4^aA^
Peleg	a_01_ = –5.9 × 10^–5^ K^–1^ a_02_ = 0.15 a_11_ = 7.9 × 10^–3^ K^–1^ a_12_ = –2.2 a_21_ = 5.7 × 10^–4^ K^–1^ a_22_ = –0.2 a_31_ = 66.9 K^–1^ a_32_ = 0.06 b_1_ = –0.016% d.b b_2_ = 1.66 × 10^–3%^ d.b	[–1.9 × 10^–4^, –3.9 × 10^–5^] [0.14, 0.19] [6.8 × 10^–3^, 8.1 × 10^–3^] [–2.3, –1.8] [5.7 × 10^–4^, 5.7 × 10^–4^] [–0.2, –0.2] [66.9, 66.9] [–0.016, –0.015] [–0.016, –0.015] [9.15 × 10^–4^, 2.73 × 10^–3^]	Training 3.9 ± 0.1 Validation 3.88 ± 0.06^bA^	Training 94.8 ± 0.2 Validation 94.8 ± 0.3^bA^
Kuhn	a_1.1_ = –2.4 × 10^–4^ K^–1^ a_1.2_ = 0.07 a_2.1_ = –1.2 × 10^–3^ K^–1^ a_2.2_ = –0.46 b_1_ = –0.015% d.b b_2_ = –7.8 × 10^–4%^ d.b	[–2.7 × 10^–4^, 2.1 × 10^–4^] [0.06, 0.07] [–1.2 × 10^–3^, –1.1 × 10^–3^] [0.44, 0.48] [–0.016, –0.015] [–1.6 × 10^–3^, 5.7 × 10^–4^]	Training 4.36 ± 0.1 Validation 4.4 ± 0.2^cA^	Training 94.0 ± 0.1 Validation 94.0 ± 0.4^cA^
GAB	X_m_ = 10.2% d.b C_0_ = 1.4 × 10^–8^ K_0_ = 10.5 H_m_ = 6099 kJ kg^–1^ H_n_ = 2895 kJ kg^–1^ b_1_ = –0.016% d.b b_2_ = 0.002% d.b	[10.1, 10.2] [1.5 × 10^–8^ ,1.7 × 10^–8^] [10.5, 10.5] [6099, 6099] [2895, 2895] [–0.016, –0.015] [0.002, 0.003]	Training 3.9 ± 0.1 Validation 4.0 ± 0.1^cA^	Training 94.8 ± 0.2 Validation 94.8 ± 0.3^bA^
Caurie	a_1.1_ = –0.016 K^–1^ a_1.2_ = 2.5 a_2.1_ = 0.02 K^–1^ a_2.2_ = –5.2 b_1_ = –0.015% d.b b_2_ = –5.5 × 10^–3%^ d.b	[–0.017, –0.015] [2.2, 2.7] [0.02, 0.02] [–5.5, –4.7] [–0.016, –0.015] [–6.5 × 10^–3^, –4.6 × 10^–3^]	Training 3.5 ± 0.1 Validation 3.6 ± 0.1^dA^	Training 96.2 ± 0.1 Validation 96.1 ± 0.2^dA^
White & Eiring	a_1.1_ = 0.16 K^–1^ a_1.2_ = –38.8 a_2.1_ = –0.20 K^–1^ a_2.2_ = 55.7 b_1_ = –0.015% d.b b_2_ = –6.5 × 10^–3%^ d.b	[0.16, 0.18] [–42.0, –37.6] [–0.21, –0.19] [53.5, 60.4] [–0.016, –0.015] [–7.5 × 10^–3^, –5.5 × 10^–3^]	Training 3.5 ± 0.1 Validation 3.5 ± 0.1^dA^	Training 96.4 ± 0.1 Validation 96.3 ± 0.2^dA^
Smith	a_1.1_ = –1.3 × 10^–3^ K^–1^ a_1.2_ = 0.48 a_2.1_ = 7.2 × 10^–4^ K^–1^ a_2.2_ = –0.19 b_1_ = –0.015% d.b b_2_ = –6.4 × 10^–3%^ d.b	[–1.3 × 10^–3^, –1.2 × 10^–3^] [0.47, 0.50] [6.6 × 10^–4^, 7.84 × 10^–4^] [–0.21, –0.17] [–0.016, –0.015] [–7.3 × 10^–3^, –5.4 × 10^–3^]	Training 3.5 ± 0.1 Validation 3.6 ± 0.15^dA^	Training 96.3 ± 0.1 Validation 96.2 ± 0.2^dA^
Chung Pfost	a_1.1_ = –9.292 × 10^–4^ K^–1^ a_1.2_ = 0.4 a_2.1_ = –5.1 × 10^–4^ K^–1^ a_2.2_ = 0.14 b_1_ = –0.015% d.b b_2_ = –5.3 × 10^–3%^ d.b	[–9.5 × 10^–4^, –8.9 × 10^–4^] [0.4, 0.4] [–5.6 × 10^–4^, 4.8 × 10^–4^] [0.13, 0.15] [–0.016, –0.015] [–6.4 × 10^–3^, –4.7 × 10^–3^]	Training 3.2 ± 0.1 Validation 3.21 ± 0.12^eA^	Training 96.9 ± 0.1 Validation 96.9 ± 0.2^eA^

**Table 3:** Estimated model parameters of the trained models (75%) and their statistical results using the validation dataset (25%).

Models	Parameters	Confidence intervals (95%)	MRE (%)	R^2^_adj_ (%)
Oswin	a_1.1_ = –7.5 × 10^–4^ K^–1^ a_1.2_ = 0.34 a_2.1_ = 4.12 × 10^–3^ K^–1^ a_2.2_ = –1.14 b_1_ = –0.015% d.b b_2_ = –4.5 × 10^–3%^ d.b	[–7.8 × 10^–4^, –7.2 × 10^–4^] [0.33, 0.35] [3.84 × 10^–3^, 4.35 × 10^–3^] [–1.21, –1.06] [–0.016, –0.015] [–5.7 × 10^–3^, –4.1 × 10^–3^]	Training 3.2 ± 0.1 Validation 3.2 ± 0.1^eA^	Training 97.0 ± 0.1 Validation 97.0 ± 0.2^eA^
DLP	a_01_ = –9.2 × 10^–4^ K^–1^ a_02_ = 0.4 a_11_ = − 3.6 × 10^–4^ K^–1^ a_12_ = 0.1 a_21_ = 1.1 × 10^–4^ K^–1^ a_31_ = –1.5 × 10^–4^ K^–1^ a_32_ = 0.04 b_1_ = –0.016% d.b b_2_ = 1.5 × 10^–3%^ d.b	[–9.4 × 10^–4^, –8.6 × 10^–4^] [0.4, 0.4] [–4.1 × 10^–4^, –2.8 × 10^–4^] [0.1, 0.1] [–2.5 × 10^–4^, –3.6 × 10^–6^] [–2.4 × 10^–4^, –8.8 × 10^–5^] [0.02, 0.07] [–0.016, –0.015] [6.2 × 10^–4^, 2.4 × 10^–3^]	Training 2.9 ± 0.1 Validation 2.9 ± 0.1^fA^	Training 97.6 ± 0.1 Validation 97.5 ± 0.1^fgA^
Polynomial	a_01_ = –1.9 × 10^–3^ K^–1^ a_02_ = 0.6 a_11_ = 5.6 × 10^–3^ K^–1^ a_12_ = –1.5 a_21_ = –0.01 K^–1^ a_22_ = –3.2 a_31_ = 9.4 × 10^–3^ K^–1^ a_32_ = –2.6 b_1_ = –0.016% d.b b_2_ = 2.4 × 10^–3%^ d.b	[–2.1 × 10^–3^, –1.6 × 10^–3^] [0.6, 0.7] [3.5 × 10^–3^, 7.7 × 10^–3^] [–2.1, –0.9] [–0.02, –7 × 10^–3^] [1.7, 4.6] [5.9 × 10^–3^, 0.01] [–3.5, –1.5] [–0.016, –0.015] [1.4 × 10^–3^, 3.2 × 10^–3^]	Training 2.9 ± 0.1 Validation 2.9 ± 0.1^ghA^	Training 97.4 ± 0.1 Validation 97.3 ± 0.2^fA^
Yanniotis & Blahovec	a_01_ = 0.016 K^–1^ a_02_ = –4.4 a_11_ = 0.05 K^–1^ a_12_ = –7.2 a_21_ = –6.9 K^–1^ a_22_ = 2347.4 a_31_ = 7.0 K^–1^ a_32_ = –2406.4 b_1_ = –0.016% d.b b_2_ = 1.4 × 10^–3%^ d.b	[0.015, 0.019] [–5.3, –4.1] [0.04, 0.06] [–9.0, –4.2] [–7.5, –7.3] [2515.8, 2568.8] [7.6, 7.9] [–2668.2, –2624.3] [–0.016, –0.015] [8.5 × 10^–4^, 2.3 × 10^–3^]	Training 2.8 ± 0.1 Validation 2.8 ± 0.1^gA^	Training 97.6 ± 0.1 Validation 97.6 ± 0.1^gA^
ML	Hyperparameters	MRE (%)	R^2^_adj_ (%)	
RT	Post-pruning standard error: 0.1	Training 1.3 ± 0.1 Validation 1.4 ± 0.1^cA^	Training 98.9 ± 0.1 Validation 98.9 ± 0.1^hA^	
kNN	Number of nearest neighbor: 4	Training 1.40 ± 0.01 Validation 1.42 ± 0.01^iA^	Training 98.8 ± 0.02 Validation 98.8 ± 0.02^hA^	
RF	Number of trees: 100	Training 1.02 ± 0.01 Validation 1.03 ± 0.02^jA^	Training 99.4 ± 0.01 Validation 99.4 ± 0.02^iA^	
SVM	Kernel function: *laplacedot* Type: *nu-SVR* C: 500.5	Training 0.23 ± 0.05 Validation 0.21 ± 0.01^kA^	Training 99.8 ± 0.02 Validation 99.80 ± 0.02^jA^	

As already explained (model training and statistical validation section), the goodnesses of fit, such as MRE(%) and R^2^_adj_(%), were used as metrics to assess the predictability of trained/validated sorption and machine learning models to describe the water sorption isotherms of dried specialty coffee beans processed by wet and semidry postharvest methods.

Results were expressed as mean ± standard error. Different lowercase letters indicate statistically significant differences (95%) for each goodness-of-fit metric (MRE and R^2^_adj_) as a function of the GAB, empirical and machine learning models (Table 3); uppercase letters indicate statistically significant differences for the training/validation iterations performed.

The statistical results revealed MRE values ranging from 0.23 to 4.5% and R^2^_adj_ values between 93.7 and 99.8% for both the training and validation partitioned datasets. The results indicated a high predictability of conventional sorption models and ML techniques to describe the water sorption isotherms of coffee beans, considering the postharvest method and sorption curve. Additionally, slight differences between the MRE and R^2^_adj_ were found for the training (75%) and validation (25%) datasets. These results indicate that neither the conventional sorption models nor the ML models tended to overfit or underfit the observations of the validation dataset^44^.

## Discussion

The influence of temperature on the water sorption isotherm (Fig. 2) can be explained by the fact that, as temperature increases, water molecules become thermodynamically less stable, weakening their attractive forces and thus reducing the moisture content in food products^34^. Similar findings regarding water sorption isotherms have been reported for green coffee beans, as well as pulped and coffee cherry fruits^7,8^. Nonetheless, at a_w_ > 0.7 in the semidry process, an inverse effect of temperature was observed, where an increase in experimental temperature resulted in higher X_e_.

The crossing of water sorption isotherms at different temperatures can be attributed to the solubilization of carbohydrates, such as sugars in food products^45^. At high a_w_ levels, water molecules may act as solvents for low-molecular-weight solutes^34^.

This trend was only observed in the semidry method because the mucilaginous coating on the coffee beans, formed during this postharvest method (Fig. 1B), is rich in sugars and pectins^46^, compared to the coffee obtained by the wet method (Fig. 1A), where this layer is removed during the fermentation process^4^. The crossing of water sorption isotherms (inverse temperature effect) has also been reported in roasted specialty coffee^1^, where the authors attributed it to the solubility of sugars in the food product at higher a_w_ and temperatures. This trend has been widely reported in various agricultural products, including green and roasted yerba mate^31^, borojó fruit^13^, gum Arabic powders^13^, whole black peppercorns^43^ and dry-crystallized *Palada payasam*^20,21^.

The postharvest method also significantly influenced (*p* < 0.05) the shape of the water sorption isotherms (Fig. 2). When comparing the sorption isotherms of dried specialty coffee beans obtained through wet and semidry, the X_e_ was significantly higher for the wet processed beans at the same a_w_ and temperature. This result indicates that the mucilaginous coating restricts the hygroscopicity of beans obtained through the semidry processing method. As stated above, unlike the wet process (which involves covered green coffee beans and coffee parchment), the semidry processing method promotes the retention of an additional mucilaginous layer on the surface of the coffee beans (covered green coffee beans with coffee parchment and mucilage coating)^47^.

This layer adheres to the coffee parchment (as a semipermeable membrane), forming a thin film that limits water vapor migration from the surroundings to the internal structure of the coffee bean via diffusion mechanisms. Conversely, the absence of this mucilaginous coating on wet-processed coffee beans facilitates water sorption, leading to a higher moisture content^47^.

The statistical results revealed a high capability of both the sorption and machine learning models in the mathematical description of water sorption isotherms from the training (75%) and validation (25%) datasets. The goodness-of-fit metrics obtained for the training dataset allowed us to rank the trained models from best to worst: SVM > RF > RT > kNN > Yanniotis & Blahovec > DLP > Polynomial > Oswin > Chung-Pfost > Smith > White & Eiring > Caurie > GAB > Kuhn > Peleg and Iglesias & Chirife. However, it is highly recommended to validate the trained models before their use in real practical applications for predicting the X_e_ of coffee beans.

**Fig. 3:**
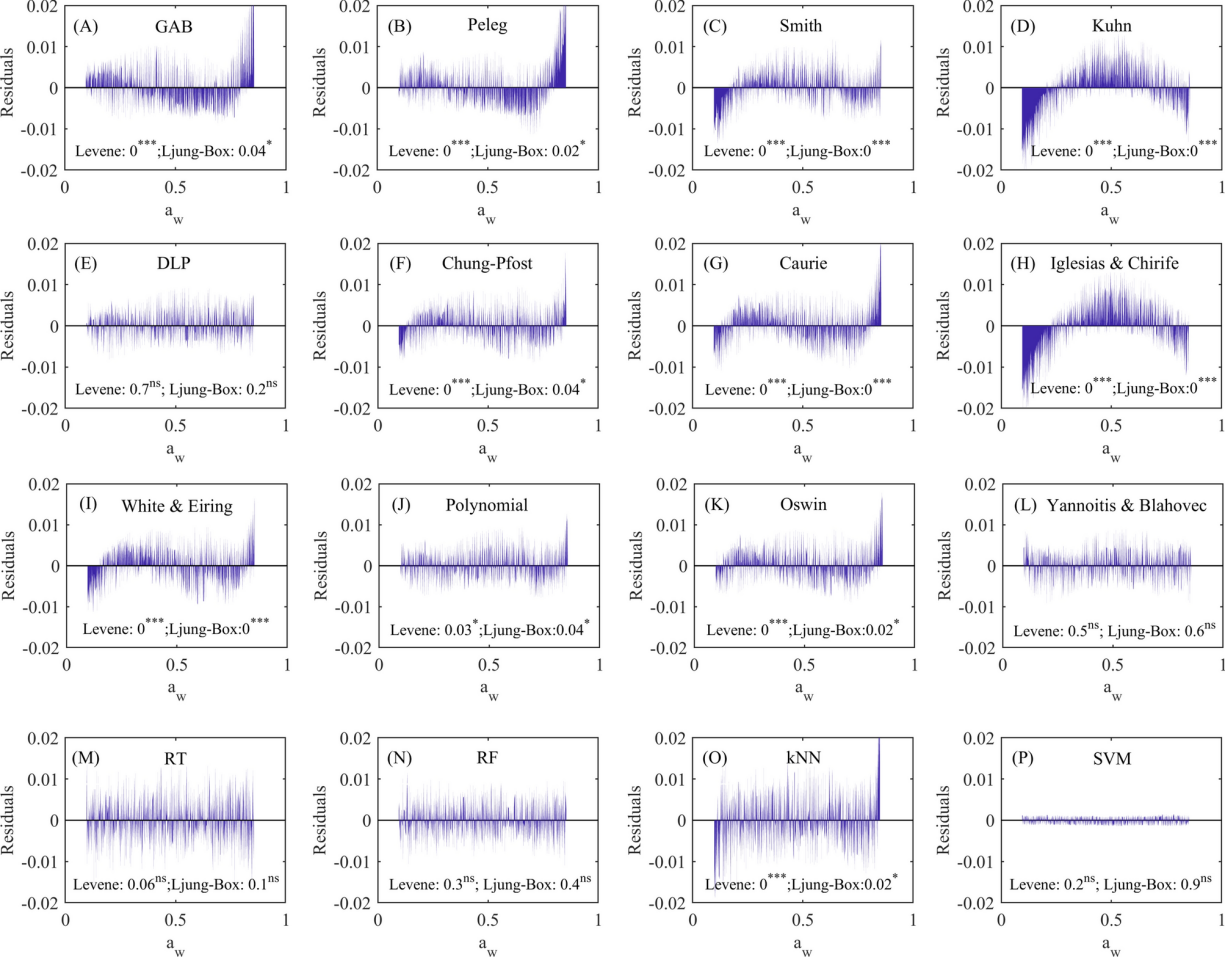
Residual validation of the trained models using the experimental training dataset (75%). ns: not significant. *p < 0.05; **p < 0.01; ***p < 0.001.

In this way, Fig. 3 depicts the statistical tests performed on the residuals obtained from the trained models. Significant nonrandom patterns (p < 0.05 according to Ljung-Box’s test) and heteroscedastic behavior (p < 0.05 according to Levene’s test) were detected in the GAB (Fig. 3A), Peleg (Fig. 3B), Smith (Fig. 3C), Kuhn (Fig. 3D), Chung-Pfost (Fig. 3F), Caurie (Fig. 3G), Iglesias & Chirife (Fig. 3H), White & Eiring (Fig. 3I), Polynomial (Fig. 3J), Oswin (Fig. 3K), and kNN (Fig. 3O). These results suggest that the residuals of these models exhibit a strong autocorrelation structure and scatter effects, indicating that predictions made using these models could underestimate and/or overestimate X_e_ as a_w_ progresses (for the training data set), and that the error variance is not constant across different aw levels. On the other hand, the residuals of the DLP, Yanniotis & Blahovec, RT, RF, and SVM models were random and homoscedastic (p > 0.05), indicating that these models’ residuals had constant variance and varied randomly as a function of aw. Therefore, any lack of compliance with either of the two residual assumptions (independence and homoscedasticity) invalidates the use of a mathematical model for practical purposes. Thus, based on the residual analysis, the use of DLP, Yanniotis & Blahovec, RT, RF, and SVM to describe the water sorption isotherms was validated due to the fulfillment of the residual hypotheses.

The parameters of the DLP and Yanniotis & Blahovec models and their confidence intervals were statistically significant (95%, Table 3). This indicates that their estimations were precise and validates the use of these parameters for understanding the water sorption process. In this sense, by using the two parameters associated with dummy variables, it was possible to quantify the differences in X_e_ between the desorption and adsorption isotherms, as well as the differences in the isotherms of coffee processed via wet and semidry methods.

Results were expressed as mean ± standard error. Different lowercase letters indicate statistically significant differences (95%) for each goodness-of-fit metric (MRE and R^2^_adj_) as a function of the GAB, empirical models (Table 2) and machine learning techniques; uppercase letters indicate statistically significant differences for the training/validation iterations performed.

As previously stated in the section on GAB and empirical models, the b_i_ parameters associated with each dummy variable quantify the increases or decreases in X_e_ between the reference (D_i_ = 0) and the actual (D_i_ = 1). The b_1_ parameter of the DLP and Yanniotis & Blahovec models was negative (Table 3), indicating that the X_e_ values in the water isotherms of the semidry method were lower than those in the wet process, as shown in Fig. 2. Similarly, both b_2_ parameters were positive, indicating that the X_e_ values from the adsorption curves were higher than those from the desorption curves (Fig. 2).

Despite the fact that the trained GAB, Peleg, Smith, Kuhn, Chung-Pfost, Caurie, Iglesias & Chirife, White & Eiring, Polynomial, Oswin, and kNN models did not fulfill the residual hypotheses (Fig. 3), we tested these models, along with those that were residually validated, to describe the validation dataset (25%).

The use of multifactor ANOVA model coupled with the LSD test were essential in selecting the most appropriate model for describing the validation (25%) dataset. The pairwise mean comparisons based on MRE and R^2^_adj_ revealed significant differences (p < 0.05) between the trained models, while the partition of experimental data set did not yield statistically significant results (*p* > 0.05). This indicates that the model selection was more sensitive to the goodness-of-fit metrics than to the number of training-iterations performed.

Using the LSD intervals, the models were ranked as follows (Tables 2 and 3): SVM > RF > RT > kNN > Yanniotis & Blahovec > DLP > Polynomial > Oswin > Chung-Pfost > Smith > White & Eiring > Caurie > GAB > Kuhn > Peleg and Iglesias & Chirife. The independent homogeneous groups identified via LSD intervals offered robust statistical criteria for model selection, prioritizing models that minimized MRE and maximized R^2^_adj_. This statistical approach confirmed that the SVM model was the most suitable for accurately describing the relationship between a_w_, temperature, and X_e_ in specialty coffee beans. With an exceptional fit (R^2^_adj_ = 99.8 ± 0.02% and MRE = 0.21 ± 0.01%), SVM clearly outperformed the other models and was validated as the optimal tool for describing the effects of postharvest methods.

However, it is also important to highlight that both the DLP and Yanniotis & Blahovec models showed a high predictive performance (DLP: R^2^_adj_ = 97.5 ± 0.1% and MRE = 2.9 ± 0.1%; Yanniotis & Blahovec: R^2^_adj_ = 97.6 ± 0.1% and MRE = 2.8 ± 0.1%). Although they were not as precise as the SVM model, their performance remained within acceptable ranges, confirming their reliability as tools for practical applications where simplicity and robustness might be more important than the highest possible fit.

**Fig. 4:**
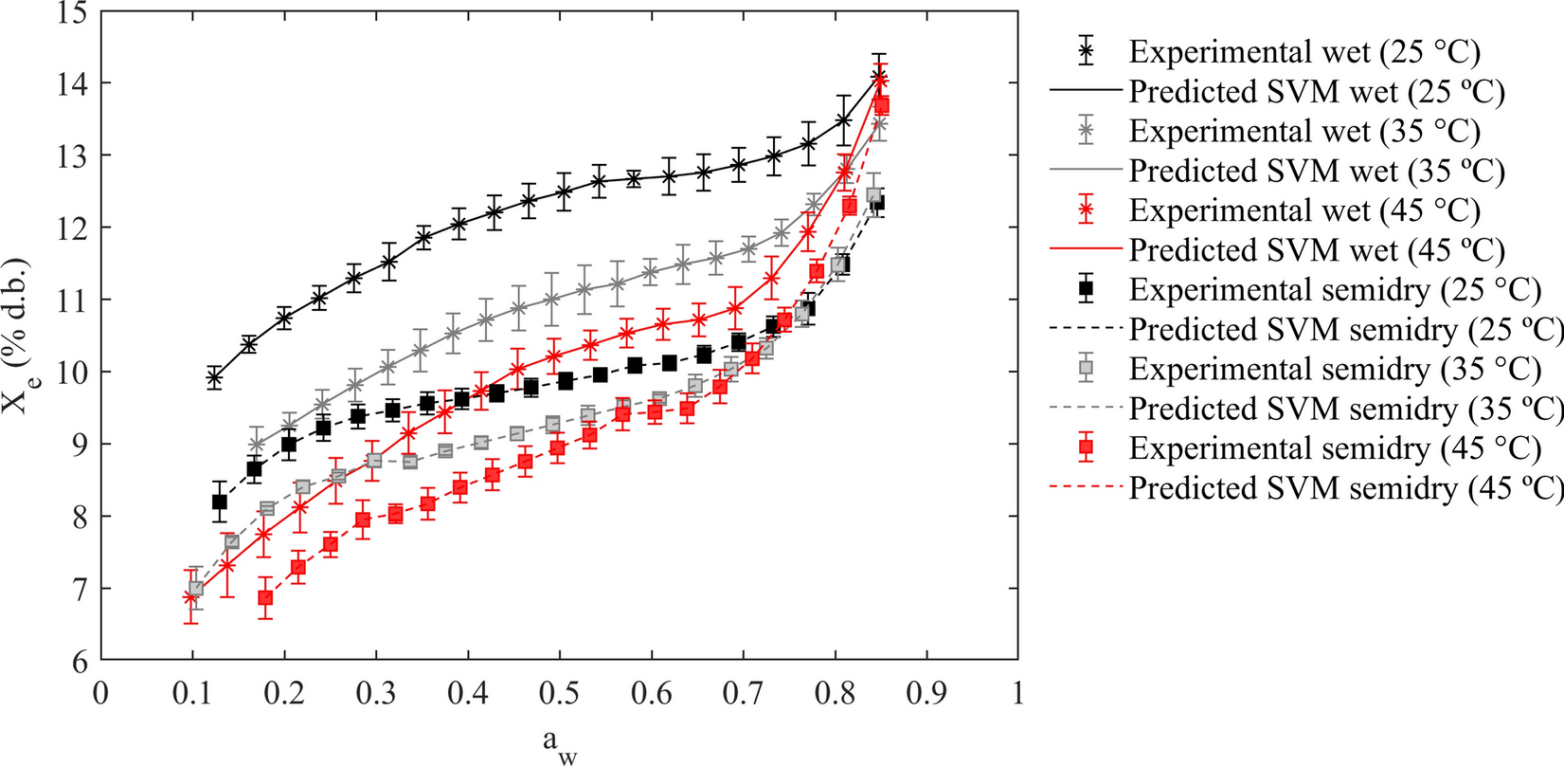
Experimental water sorption isotherms and predicted values via the trained/validated SVM model for specialty coffee beans obtained by different postharvest treatments at temperatures of 25, 35, and 45 °C.

Finally, to illustrate the greatest goodness of fit of the trained/validated SVM model, Fig. 4 depicted the experimental sorption isotherms (mean ± standard deviation) as a function of temperature and postharvest method. As shown in Fig. 4, the SVM technique successfully modeled the water sorption isotherms, demonstrating its ability to accurately describe the type II sorption shape, as well as the influence of temperature and postharvest method on X_e_. The model effectively reflected the progression of the X_e_ in relation to a_w_ and temperature (ranging from 0.1 to 0.85 and from 25 to 45 °C), and it successfully described the inverse temperature effect in the semidry method at high a_w_ levels, validating its use for accurately predicting the moisture content of coffee beans processed by different postharvest methods under a wide range of environmental storage conditions.

The feasibility of using SVM for mathematical modeling of sorption isotherms has been demonstrated in various food products, including Achira biscuits^18^ and coffee cherry beans^17^. In both studies, the authors reported that the SVM was the most robust model for describing the influence of temperature and a_w_ on X_e_.

This work is a first step in developing data-driven machine learning models to describe water sorption isotherms in dried specialty coffee beans processed by different postharvest methods. Future research should explore the influence of temperature and moisture on sensory quality attributes and bioactive compounds in coffee beans and integrate this information into robust machine learning models to improve the storage management of coffee at an industrial level.

## Conclusions

The water sorption behavior of specialty coffee beans processed by wet and semidry postharvest methods exhibited a characteristic type II S-shaped curve, typical of macroporous food products. The postharvest processing method significantly influenced the hygroscopic properties of the coffee beans, resulting in distinct S-sigmoid curve shapes between the wet and semidry methods. Semidry coffee exhibited lower moisture content values at the same water activity and temperature compared to the wet method and showed an inverse temperature effect at water activity levels higher than 0.7. This effect may be attributed to possible sugar dissolution at high water activity levels and the adsorbate-adsorbent interactions in semidry coffee beans. From the experimental analysis of the water sorption isotherms, it was evident that the mucilaginous coating found in semidry coffee beans played a protective role against water sorption, as indicated by the lower moisture content values observed across the range of water activities and temperatures studied.

Mathematical modeling of water sorption isotherms, considering postharvest processing methods, was conducted using various conventional models and machine learning techniques. Among the models tested, the SVM provided the most accurate fit for describing the isotherms of dried specialty coffee beans. This mathematical tool could prove valuable for future applications in real-time monitoring of moisture content in dried specialty coffee beans processed by wet and semidry methods, based on their storage conditions. Further research should focus on implementing the calibrated SVM technique at the industrial level as a virtual representation of the storage process, facilitating real-time decision-making to enhance coffee quality management during storage.

## Data Availability

The datasets generated and/or analysed during the current study are available in the [Experimental water sorption isotherms and mid-infrared spectra of parchment coffee beans processed by wet and semi-dry postharvest methods] repository, [10.17632/pffnhth7xd.1].
